# Predictors of isolated systolic hypertension among type 2 diabetes mellitus patients in Jimma University Specialized Hospital, Southwest Ethiopia

**DOI:** 10.1186/s13104-019-4550-3

**Published:** 2019-08-15

**Authors:** Baye Dagnew, Yigizie Yeshaw

**Affiliations:** Department of Human Physiology, University of Gondar, P.O. Box 196, Gondar, Ethiopia

**Keywords:** Cross-sectional, Hypertension, Isolated systolic hypertension, T2DM, Ethiopia

## Abstract

**Objectives:**

Systolic blood pressure rise among T2DM patients was main risk factor for cardiovascular disease. Objective of this study was to identify predictors of isolated systolic hypertension among T2DM patients at Jimma University Specialized Hospital, 2016. We conducted cross sectional study using simple random sampling and interviewer administered questionnaire. Isolated systolic hypertension is SBP ≥ 140 mmHg and < 90 mmHg. Data entered and analyzed using Epi Data and SPSS respectively. Predictor factor was declared at p < 0.05.

**Results:**

A total of 315 T2DM took part. Prevalence of ISH was 27.6% [95% CI (22.7, 32.5%)]. One hundred sixty and two (51.4%) patients were males with mean age of 54.1 from 22 to 87 years. Male sex [AOR = 2.4, 95% CI 1.21–4.72, p = 0.012], unemployment [AOR = 3.22, 95% CI 1.48–7.03, p = 0.003], age of 47–55 [AOR = 2.63, 95% CI 1.03–6.70, p = 0.044], single [AOR = 2.26, 95% CI 1.13–4.51, p = 0.021], ≤ Grade 8 [AOR = 2.94, 95% CI 1.10–7.85, p = 0.03] and income (ETB) 501–800 [AOR = 21.9, 95% CI 7.62–63.1, p < 0.001], 801–1500 [AOR = 5.78, 95% CI 2.55–13.1, p < 0.001] and > 1500 [AOR = 4.23, 95% CI 1.74–10.30, p = 0.001] were significant factors of ISH. The health sector has to establish preventive strategies for ISH among T2DM patients by giving special attention to predictor factors.

**Electronic supplementary material:**

The online version of this article (10.1186/s13104-019-4550-3) contains supplementary material, which is available to authorized users.

## Introduction

Hypertension is the common comorbidity of diabetes mellitus (DM) [1]. It is prevalent in developing countries [2], major risk for coronary disease [3]. Isolated systolic hypertension (ISH) is common form of hypertension bringing morbidity and mortality mainly among elderly [4]. ISH is SBP ≥ 140 mmHg and DBP < 90 mmHg. According to JNC-VI; grade 1 ISH (SBP < 160 mmHg), grade 2 (SBP < 180 mmHg) and grade 3 (SBP ≥ 180 mmHg) [5]. Raised SBP among T2DM patients was the major risk factor for cardiovascular diseases [6]. Diabetes patients experienced high vascular resistance explained by vascular remodeling and increased fluid volume related to hyperglycemia [7]. Vascular problems are explained by hypertension among T2DM [8].

Decreasing of SBP by 10 mmHg lead to 12% reductions of diabetes complications [9, 10]. SBP increases after 50–60 years due to age-related changes in vasculature [11]. ISH mainly affects those 50 years and above [12]. Arterial stiffness is accountable for pathogenesis of hypertension [13, 14]. It is common in DM patients caused by endothelial dysfunction leading to inflammation [15, 16]. ISH is associated with ageing leading to morbidity and mortality due to cerebrovascular disturbance [4]. There was 85.6% prevalence of hypertension among DM patients in Benghazi [17]. ISH ranges from 20.3% in primary care patients to 35% in general population [18]. A study in Saudi revealed 7.6% of ISH among adults associated with education, obesity, DM, and dyslipidemia [19]. Prevalence of 43.6% was reported in Nigerian adults associated with increased age [20]. ISH is common in obese, males, low education level and smokers [21]. A study in Ghana revealed 37.4% ISH which was predicted by patient’s age [22]. In DM patients, ISH is associated with older age, male sex, DM duration and overweight [17]. For 5th–6th decades of life SBP and DBP increased with age and onwards SBP continues to increase [23, 24]. There were limited studies globally and none was found in Ethiopia on predictors of ISH. So this study aimed to identify predictors of ISH among T2DM, to add information to scientific community and put baseline for Ethiopia.

## Main text

### Patients and methods

#### Study setting

We conducted the study at Jimma University Specialized Hospital (JUSH), Jimma, 352 km away from Addis Ababa. We found 1853 T2DM patients (source population) in medical records at JUSH immediately before starting data collection of which 850 patients were accessible during data collection.

Study design: Institution-based cross-sectional study design was employed.

Study period: It was conducted from April 01 to July 30, 2016.

#### Sample size determination and sampling technique

Sample size was calculated using Raosoft sample size calculator [25] assuming 95% level of confidence, 5% margin of error, population of 1853 and p = 37.4% [22]. We had 302 samples and after adding 5% oversampling to account for any unpredictable events final sample size was 318. Computer generated simple random sampling was used to select patients using their medical registration number.

#### Eligibility criteria

All T2DM patients aged ≥ 20 years who were present during study period were included whereas those T2DM patients who were severely ill and pregnant women were excluded from the study.

#### Variables of the study

Dependent variable: Isolated systolic hypertension.

Independent variables: Age, sex, residence, marital status, lifetime khat chewing, lifetime cigarette smoking, lifetime alcohol drink, income, educational level, treatment options for diabetes, BMI, duration of DM, Fasting plasma glucose, occupation,

#### Operational definitions

Isolated systolic hypertension (ISH): SBP ≥ 140 mmHg and DBP < 90 mmHg [5, 26].

Hypertension (HTN): SBP and/or DBP of 140/90 mmHg or greater. The 2007 ESH/ESC recommended two distinct BP targets; 140/90 in low moderate risk hypertensive and 130/80 mmHg in high-risk hypertensive [27].

Isolated diastolic hypertension (IDH): DBP ≥ 90 mmHg and SBP < 140 mmHg.

Mean arterial pressure (MAP): The sum of DBP and one-third of the pulse pressure. MAP ranges 70–106.7 mmHg for average adults.

Pulse pressure (PP): The difference between systolic and diastolic pressure which ranges from 30 to 50 mmHg in average adults.

Fasting plasma glucose (FPG) level: Plasma glucose level when expressed in mg/dl of blood which is measured by taking blood sample from a person who was not taking anything orally for at least 8-h prior to taking the blood sample.

Body mass index: A person with BMI of < 18.5 kg/m^2^, 18.5–24.9 kg/m^2^, and > 24.9 kg/m^2^ is considered as underweight, normoweight and overweight respectively.

*Rural residence* Settling in country side outside of big cities or towns in Ethiopia are referred as rural residents whereas those living in big cities (like Jimma town) were urban residents [28].

*Lifetime khat chewing* Use of Khat at least once in an individual’s lifetime [29].

*Lifetime cigarette smoking* Use of cigarette smoke at least once in an individual’s lifetime [30].

*Lifetime alcohol drink* Drinking of alcohol beverage at least once in an individual’s lifetime [31].

*Hypoglycaemia* An event during which typical symptoms of hypoglycemia are accompanied by a measured plasma glucose concentration ≤ 70 mg/dl [32].

#### Data collection instrument and procedure

A pre-tested (17 T2DM patients at Shenen Gibe Hospital.) interviewer administered questionnaire was used to collect data. Questionnaire was adapted from previous published articles [3, 7, 10, 11, 13, 22, 33–35], prepared in English and translated to Amharic and retranslated to English by another person (see Additional file 1). Four data collectors and two supervisors participated in data collection. Data were extracted from patient charts. Fasting plasma glucose was determined using glucometer.

Blood pressure was measured in sitting position. The arm which was used for BP measurement was supported on flat table and using mercury sphygmomanometer BP cuff with the appropriate cuff size that covers two-thirds of the arm. Before measurement, participants were asked to rest for at least 5 min and if they were taking any caffeinated beverages they were rested for 30 min. Two consecutive measurements had been taken in 5 min gap before noon and average was taken for each.

#### Data quality management/control

Orientation was given for supervisors and data collectors about content of tool, data extraction and interview technique, ethical issues and measurement techniques for 6 h before starting data collection. Handy supervision and completeness of questionnaire were done by supervisors and principal investigator on daily basis.

#### Data processing and analysis

Epi Data-3.1 and SPSS-21 were used for data entry and analysis respectively. Crude association of independent variables with ISH was performed at p < 0.2. Variables in bivariable analysis with p < 0.2 were candidates for multivariable logistic regression. Both crude odds ratio (COR) and adjusted odds ratio (AOR) with 95% confidence interval (CI) were computed to demonstrate strength of association. In multivariable logistic regression, variables with p < 0.05 were declared as statistically significant. The null hypothesis was rejected if p > 0.05, CI includes 1 and if OR is 1.

### Results

#### Sociodemographic and clinical records of study participants

Three hundred fifteen T2DM patients participated in the study. The mean age of study participants was 54.1 (± 11.96) ranging from 22 to 87 years. One hundred sixty and two (51.4%) study participants were males, 165 (52.4%) individuals were Muslim. Two hundred and one (63.8%) respondents were ≤ grade 8. Sixty-three (20%) individuals had encountered episodes of hypoglycemia. Two hundred and ten patients (66.7%) relied on oral hypoglycemic agents as treatment option for T2DM. Fasting plasma glucose was 126 mg/dl and above among 216 (68.6%) of patients (Table 1).

**Table 1:** Sociodemographic and clinical profiles of study participants (n = 315), Jimma, Southwest Ethiopia, 2016

Variable	Categories	Frequency	Percent
Age (in years)	22–46	81	25.7
	47–55	84	26.7
	56–62	81	25.7
	63–87	69	21.9
Sex	Male	162	51.4
	Female	153	48.6
Religion	Orthodox	117	37.1
	Muslim	165	52.4
	Protestant	21	6.7
	Catholic	12	3.8
Marital status	Married	138	43.8
	Single	177	56.2
Educational level	Grade 8 and lower	201	63.8
	Grade 9–12	75	23.8
	College and above	39	12.4
Residence	Urban	222	70.5
	Rural	93	29.5
Episodes of hypoglycemia	Yes	63	20.0
	No	252	80.0
Treatment option for DM	Insulin only	30	9.5
	OHA	210	66.7
	Both insulin and OHA	75	23.8
Lifetime khat use	Yes	165	52.4
	No	150	47.6
Lifetime alcohol drink	Yes	99	31.4
	No	216	68.6
Lifetime cigarette smoking	Yes	30	9.5
	No	285	90.5
BMI	Underweight	15	4.8
	Normoweight	183	58.1
	Overweight	117	37.1
Duration of DM (years)	≤ 7	207	65.7
	> 7	108	34.3
FBS (mg/dl)	≤ 125	99	31.4
	≥ 126	216	68.6
Monthly income (in ETB)	Below 501	98	31.1
	501–800	66	21.0
	801–1500	78	24.8
	> 1500	73	23.2
Occupation	Employed	114	36.2
	Unemployed	201	63.8

*OHA* oral hypoglycemic agent, *BMI* body mass index, *FBS* fasting blood sugar, *ETB* Ethiopian Birr, *DM* diabetes mellitus

#### Prevalence of isolated systolic hypertension and vascular parameters

The prevalence of ISH was 27.6% [95% CI (22.7, 32.5%)] with a mean of 124.4 ± 18.95 mmHg. The overall comorbid hypertension was 41% with mean systolic to a diastolic blood pressure ratio of 1.62 ± (1.56) (Table 2).

**Table 2:** Blood pressure profiles of study participants (n = 315), Jimma, Southwest Ethiopia, 2016

BP profile	Category	Frequency	Percent	Mean ± SD
Isolated systolic HTN	Yes	87	27.6	
	No	228	72.4	
Overall hypertension	Yes	129	41	
	No	186	59	
Isolated diastolic HTN	Yes	42	13.3	
	No	273	86.7	
SBP to DBP ratio	Minimum 1.11	Maximum 2.5	–	1.62 ± 1.56
Mean arterial pressure	Minimum 63.3	Maximum 120	–	93.2 ± 11.9
Pulse pressure	Minimum 10	Maximum 100	–	46.9 ± 40

*SD* standard deviation, *HTN* hypertension, *SBP* systolic blood pressure, *DBP* diastolic blood pressure

Sex, BMI, employment status, marital status, FPG, patients’ age, monthly income, lifetime alcohol drink, and educational level were candidates for final model. Male sex, older age, employment, educational level, income, and marital status were significantly associated with ISH. The odds of ISH among T2DM patients was 2.4 times higher [AOR = 2.4, 95% CI 1.21–4.72] in males than females. Patients aged 47–55 years had odds of 2.6 [AOR = 2.63, 95% CI 1.03–6.70] as compared to those aged 22–46 years. The odds of having ISH was 3.2 times among employed patients [AOR = 3.22, 95% CI 1.48–7.03] relative to unemployed T2DM patients. Patients who attained education level of ≤ grade 8 had odds of 2.9 [AOR = 2.94, 95% CI 1.10–7.85] as compared to those attaining college and above. Regarding marital status, single T2DM patients were 2.26 times more likely to get ISH than [AOR = 2.26, 95% CI 1.13–4.51] married individuals (Table 3).

**Table 3:** Predictors of isolated systolic hypertension among type 2 diabetes mellitus patients in bivariable and logistic regression analysis (n = 315), Jimma, Southwest Ethiopia, 2016

Variables	Isolated systolic hypertension		OR with 95% CI	
	Yes (%)	No (%)	COR	AOR
Sex				
Male	33 (20.4)	129 (79.6)	2.1 (1.29–3.54)	2.4 (1.21–4.72)*
Female	54 (35.3)	99 (64.7)	1	1
BMI				
Normoweight	63 (31.8)	135 (68.2)	1	1
Overweight	24 (20.5)	93 (79.5)	1.81 (1.06–3.10)	1.97 (0.97–4.00)
Occupation				
Employed	24 (21.1)	90 (78.9)	1.71 (0.99–2.94)	3.22 (1.48–7.03)**
Unemployed	63 (31.3)	138 (68.7)	1	1
Marital status				
Married	51 (37)	87 (63)	1	1
Single	36 (20.3)	141 (79.7)	2.29 (1.39–3.79)	2.26 (1.13–4.51)*
FPG (mg/dl)				
≤ 125	36 (36.4)	63 (63.6)	1	
≥ 126	51 (23.6)	165 (76.4)	1.85 (1.10–3.09)	–
Age in years				
22–46	21 (25.9)	60 (74.1)	1	1
47–55	13 (15.5)	71 (84.5)	1.91 (0.88–4.14)	2.63 (1.03–6.70)*
56–62	19 (23.5)	62 (76.5)	1.14 (0.56–2.33)	0.94 (0.39–2.25)
≥ 63	34 (49.3)	35 (50.7)	0.36 (0.18–0.72)	0.32 (0.13–0.78)*
Monthly income in ETB				
< 501	52 (53.1)	46 (46.9)	1	1
501–800	6 (9.1)	60 (90.9)	11.3 (4.47–28.6)	21.9 (7.62–63.1)***
801–1500	18 (23.1)	60 (76.9)	3.77 (1.95–7.29)	5.78 (2.55–13.1)***
> 1500	11 (15.1)	62 (84.9)	6.37 (2.99–13.54)	4.23 (1.74–10.30)***
Educational status				
≤ Grade 8	48 (23.9)	153 (76.1)	1.99 (0.97–4.10)	2.94 (1.10–7.85)*
Grade 9–12	24 (32)	51 (68)	1.33 (0.59–2.98)	0.79 (0.27–2.28)
≥ College	15 (38.5)	24 (61.5)	1	1

* Significant (p < 0.05)

** Significant (p < 0.01)

*** Significant (p < 0.001)

### Discussion

The current study, the first in Ethiopia in its kind, revealed 27.6% with 95% CI (22.7, 32.5%) prevalence of ISH. The high prevalence of ISH in type 2 diabetes mellitus patients might be related with increased vascular resistance via remodeling of blood vessels following hyperglycemia [7]. This finding is higher than a finding from Hosanna (8.6%) [36], though this study was not purposely done to determine ISH rather it was to determine overall hypertension. This is also lower than a study in Sweden with 6% systolic hypertension in diabetes patients [37]. This difference might be due to differences in sample size, adherence to pharmacologic treatment and self-care behavior and duration of disease across different areas. Nonetheless, the prevalence of ISH in the current study is lower than previous study conducted in Ghana, western Africa (37.4%) [22]. The possible difference might be linked with variations in the management of DM and socioeconomic factors of study participants.

In the final model significantly associated factors of ISH among T2DM patients were male sex, older age, employment status, lower educational level, monthly income, and marital status.

The odds of ISH among T2DM male patients was 2.4 times higher than females. This is similar to a study in South Africa [38] and Benghazi [17, 38]. However, there are reports against our study finding [39]. The possible reason for males to get ISH could be due to males have higher cardiac output than females. Another possible physiological reason might be because females have more estrogen and progesterone which decreases plasma cholesterol and vascular resistance respectively.

Participants with age of 47–55 years were 2.6 times more likely to get ISH than aged 22–46 years. It is in line with previous study [12, 40]. The possible reason for this might be atherosclerosis is common in older age due to vascular deposition of calcium and fat [41]. Employed patients were 3.2 times more likely to develop ISH than unemployed patients which is in line with other study [42]. Association of employment with ISH might be because employed individuals have sedentary lifestyle and unemployed individuals may be engaged in physical activities for searching out daily work but aforementioned associations are discouraged by other studies [17, 38]. Educational status of ≤ grade 8 were 2.9 times more likely to develop ISH than those in College and above level. This finding is similar with a study in Australia which reported that, decreased education level was associated with increased hypertension [34], Boston [33] and Saudi [19]. The probable reason for higher ISH in lower education level might be lack of awareness about lifestyle modification among patients who attained lower educational level. The odds of ISH in patients with monthly income of 501–800ETB, 801–1500ETB, and > 1500 ETB was 21.9 times, 5.78 times, and 4.23 times than those with monthly income of ≤ 500ETB respectively. This is consistence with previous studies [43–45]. This might be due to individuals with higher income are more likely to be obese and have sedentary lifestyle. Single T2DM patients were 2.26 times more likely to acquire ISH than married patients. This might be due to possible stress which lead to increase stress hormones in single than married patients. However, this finding is against previous study [36]. Body mass index and duration of DM were associated with hypertension in some studies [46] but it was not significant in our study.

### Conclusion

In this study ISH was major public health problem. Being male, single, older age, lower education level and income were significant predictors. The findings of this study indicated health sector stakeholders to establish preventive strategies for ISH among T2DM patients by giving more attention to above mentioned predictor factors.

## Limitations of the study

The study cannot show cause-effect relationship. Social desirability and recall bias were also possible limitations.

## Additional file

**Additional file 1.** English version questionnaire to assess predictors of isolated systolic hypertension among type 2 DM patients.

## Data Availability

The dataset in the current study is available from the corresponding author upon request.

## Abbreviations

AOR: adjusted odds ratio
BMI: body mass index
CI: confidence interval
COR: crude odds ratio
DBP: diastolic blood pressure
DM: diabetes mellitus
EPI Data: Epidemiological Data
FPG: fasting plasma glucose
HTN: hypertension
IDH: isolated diastolic hypertension
ISH: isolated systolic hypertension
JNC: Joint National Committee on Prevention, Detection. Evaluation and treatment of high blood pressure
JUSH: Jimma University Specialized Hospital
SBP: systolic blood pressure
SPSS: Statistical Package for Social Sciences
