# Supplementary material for: Predictors of isolated systolic hypertension among type 2 diabetes mellitus patients in Jimma University Specialized Hospital, Southwest Ethiopia
Source: BMC Res Notes. 2019 Aug 15;12:510. doi: 10.1186/s13104-019-4550-3 (PMC6694682; doi:10.1186/s13104-019-4550-3)
Supplement: Supplementary file 1 — Additional file 1. English version questionnaire to assess predictors of isolated systolic hypertension among type 2 DM patients. [file 13104_2019_4550_MOESM1_ESM.docx]

**English version questionnaire to assess predictors of isolated systolic hypertension among type 2 DM patients**

**Questionnaire code _____________**

**Instruction to data collectors: Please fill (for open ended questions) or encircle (for alternatives) the responses of participants based on the nature of questions**

**Part I- Sociodemographic characteristics**

| S/N | Variables | **Response (alternatives)** |
| --- | --- | --- |
| 101 | Age | ____________ years |
| 102 | Sex | 0. Male  1. Female |
| 103 | Religion | 0. Orthodox  1.Muslim  2.Protestant  3.Catholic  4. Others(specify) |
| 104 | Ethnicity | 0.Oromo  1.Amhara  2.Tigre  3.Guraghe  4 .others(specify) |
| 105 | Educational status | 0. Grade 8 and lower  1. Grade 9-12  **2**. College and above |
| 106 | Marital status | 0. Single  1. married  2.divorced  3.widowed |
| 107 | Occupation | 0. Government employee  1. Private employee  2. Merchant  3. Farmer  4. House wife  5. Daily laborers  6.no job  7. Others specify-- |
| 108 | Monthly Income | _______________________Ethiopian birr |
| 109 | Residence | 0. Urban  1. Rural |

**Part II- Medical history - see chart of the patient**

| 201 | Episodes of hypoglycemia | 0. Yes  1. No |
| --- | --- | --- |
| 202 | Type of DM treatment the patient rely on | 0. Insulin  1.Oralhypoglycemic drugs  2.Both |
| 203 | Duration of DM (disease) since diagnosis | ______ Months |
| 204 | Fasting plasma glucose level | ____________mg/dl |

**Part III- Substance use assessment (alcohol intake, khat chewing, cigarette smoking)**

| 301 | Have you ever chewed khat in your life time? | 0. Yes  1. No |
| --- | --- | --- |
| 306 | Have you ever drunk alcohol in your life time? | 0. Yes  1. No |
| 311 | Have you ever smoked cigarette in your life time? | 0. Yes  1. No |

**Part IV- Physical examination (measurement)**

| S/N | Variable | Response | Remark |
| --- | --- | --- | --- |
| 401. | Weight in kg | ___________ |  |
| 402 | Height in cm | ___________ |  |
| 403 | Systolic blood pressure in mmHg | ___________ |  |
| 404 | Diastolic blood pressure in mmHg | ____________ |  |

**Thank you again for your participation**
